# Urinary incontinence among older adults in Saudi Arabia: prevalence, severity, and associated factors

**DOI:** 10.1186/s12877-026-08013-5

**Published:** 2026-07-25

**Authors:** Ageel Alharbi, Saleh AlRamadan, Faisal Alhejazi, Hend Almarshad, Yara Aljomah, Aseel Alwahhabi, Remaz Alghamdi

**Affiliations:** 1 King Abdulaziz Medical City, National Guard Health Affairs, Riyadh, Saudi Arabia; 2 Imam Abdulrahman bin Faisal National Guard Hospital, Dammam, Saudi Arabia; 3 King Saud bin Abdulaziz University for Health Sciences, Riyadh, Saudi Arabia

**Keywords:** Outpatient clinics, Urinary incontinence, Urinary tract infection, Benign prostatic hyperplasia, Saudi Arabia, Internal medicine, Geriatric, Urinary incontinence

## Abstract

**Background:**

Urinary incontinence (UI) is a common yet under-recognized condition among older adults, particularly in outpatient settings, where symptoms are often underreported. Despite its significant impact on quality of life, data on the prevalence and clinical factors associated with UI in Saudi Arabian outpatient populations remain limited.

**Method:**

A cross-sectional study was conducted among 290 adults aged > = 60 years attending NGHA outpatient clinics in Riyadh and Dammam, Saudi Arabia. Participants were recruited consecutively during the study period. Urinary incontinence was assessed using the validated Arabic version of the International Consultation on Incontinence Questionnaire - Short Form (ICIQ-SF), and multivariable logistic regression analysis was performed to identify factors independently associated with urinary incontinence.

**Result:**

Urinary incontinence was reported in 40.3% of participants and was more common among women than men. In multivariable analysis, a history of urinary tract infection was strongly associated with urinary incontinence (AOR 3.73; 95% CI 2.19–6.35; *p* < 0.001), while benign prostatic hyperplasia was independently associated with urinary incontinence among men (AOR 2.81; 95% CI 1.05–7.49; *p* = 0.039). Other demographic and clinical variables were not independently associated with urinary incontinence.

**Conclusion:**

Urinary incontinence is common among older adults attending outpatient clinics, with urinary tract infection and benign prostatic hyperplasia identified as key associated factors.

## Introduction

Urinary incontinence (UI) is a common and often under-recognized condition among older adults, despite its well-established impact on physical functioning, psychological well-being, quality of life, and daily activities. Many older individuals perceive urinary incontinence as a normal consequence of aging or feel reluctant to discuss symptoms due to embarrassment [1, 2]. This is particularly evident in busy outpatient clinics, where urinary incontinence may not be systematically screened, and patients may not voluntarily report symptoms. Consequently, under recognition of urinary incontinence can mask a substantial clinical burden and delay in identification and management of a potentially treatable condition [3].

International studies have reported that the prevalence of urinary incontinence among older adults ranges from 20% to 50%, with considerable variability depending on age, sex, and study setting [3, 4]. Increasing age, female sex, functional impairment, reduced mobility, chronic medical conditions such as diabetes and obesity, and the use of certain medications have been consistently identified as factors associated with urinary incontinence [2, 5, 6].

Despite the rapidly growing older population in Saudi Arabia, available data on urinary incontinence remain limited. Most studies conducted in the region have focused on community-based samples or specific population groups rather than patients attending outpatient clinics [7]. As a result, the findings from these studies cannot be easily applied to routine ambulatory care, leaving gaps in understanding the urinary incontinence in outpatient practice.

Therefore, this study aimed to determine the prevalence of urinary incontinence among older adults attending National Guard Health Affairs (NGHA) outpatient clinics in Riyadh and Dammam, and to identify clinical factors associated with its occurrence.

## Materials and methods

### Study design and setting

A cross-sectional study was conducted in National Guard Health Affairs (NGHA) outpatient clinics in Riyadh and Dammam. The included clinics represented multiple specialties and were not limited to geriatric or urology services. Participants were recruited consecutively from the specified outpatient clinics during the study period.

### Study period

The study was conducted from 1 August to 29 November 2025.

### Participant recruitment and sample size

Participants were recruited consecutively from the specified outpatient clinics during the study period. The sample size was estimated for a single-proportion prevalence study using an expected UI prevalence of 35% based on prior literature, a 95% confidence level, and an absolute precision of 6%, giving a minimum required sample of approximately 243 participants. The final analyzed sample of 290 participants exceeded this minimum requirement.

### Inclusion criteria

Adults aged > = 60 years attending National Guard Health Affairs outpatient clinics during the study period were eligible for inclusion.

### Exclusion criteria

Patients were excluded if they were bedbound, totally dependent in basic activities of daily living, had a diagnosis of dementia, or had an indwelling Foley catheter.

### Data collection

Data were collected by seven trained data collectors, including the principal investigator. The validated Arabic version of the International Consultation on Incontinence Questionnaire - Short Form (ICIQ-SF) was used as the core questionnaire. Additional items were included for clinical and functional assessment and to obtain information on demographics, comorbidities, medications, functional status, urinary leakage characteristics, and perceived impact on daily life.

Urinary incontinence was defined according to participant responses to the ICIǪ-SF leakage items. Urinary tract infection history was defined as a physician-diagnosed history of urinary tract infection as reported by the participant or documented in the medical record. Benign prostatic hyperplasia was defined as a physician-diagnosed condition among male participants. Mobility dependence was defined as needing assistance with ambulation or transfers. Diuretic use referred to current use at the time of data collection.

### Statistical analysis

Data were analyzed using IBM SPSS Statistics (version 26). Descriptive statistics were used to summarize baseline characteristics. Variables with *p* < 0.20 in univariable analysis were entered into a multivariable logistic regression model to identify factors independently associated with urinary incontinence. Adjusted odds ratios (AORs) with 95% confidence intervals (CIs) were reported. Statistical significance was defined as *p* < 0.05.

### Ethical approval

The study received IRB approval from the National Guard Health Affairs (NRR25/108/5). All participants provided informed consent.

## Results

### Baseline characteristics

A total of 290 older adults were included in the analysis. Most participants were between 60 and 70 years of age (73.1%), while 19.3% were aged 71–80 years. Only a small proportion were aged 81 years or older. Women accounted for 62.1% of the study population.

Chronic medical conditions were common. Hypertension (64.1%) and diabetes mellitus (58.6%) were the most frequently reported comorbidities. Approximately one-fifth of participants had chronic lung disease or asthma, and a smaller proportion reported a prior history of stroke.

Among male participants, more than one-quarter had a diagnosis of benign prostatic hyperplasia. Nearly one-quarter of the cohort reported some degree of mobility dependence. Participant characteristics are summarized in Table 1.

**Table 1:** Patient’s demographics and clinical characteristics

Variables	*n* = 290 (%)
Female	180 (62.10)
Male	110 (37.90)
Age 60–70 years	212 (73.10)
Age 71–80 years	56 (19.30)
Age 81–90 years	18 (6.20)
Age > 90 years	4 (1.40)
Hypertension	186 (64.10)
Diabetes mellitus	170 (58.60)
COPD/asthma	59 (20.30)
History of stroke	18 (6.20)
BPH	29 (26.3)
Mobility dependence	72 (24.80)

### Prevalence and severity of urinary incontinence

Overall, 40.3% of participants reported urinary incontinence. The condition was more frequently reported by women (42.8%) than men (36.4%). An increasing in urinary incontinence prevalence was observed with advancing age, with the highest rates seen among those aged 81–90 years.

**Fig. 1:**
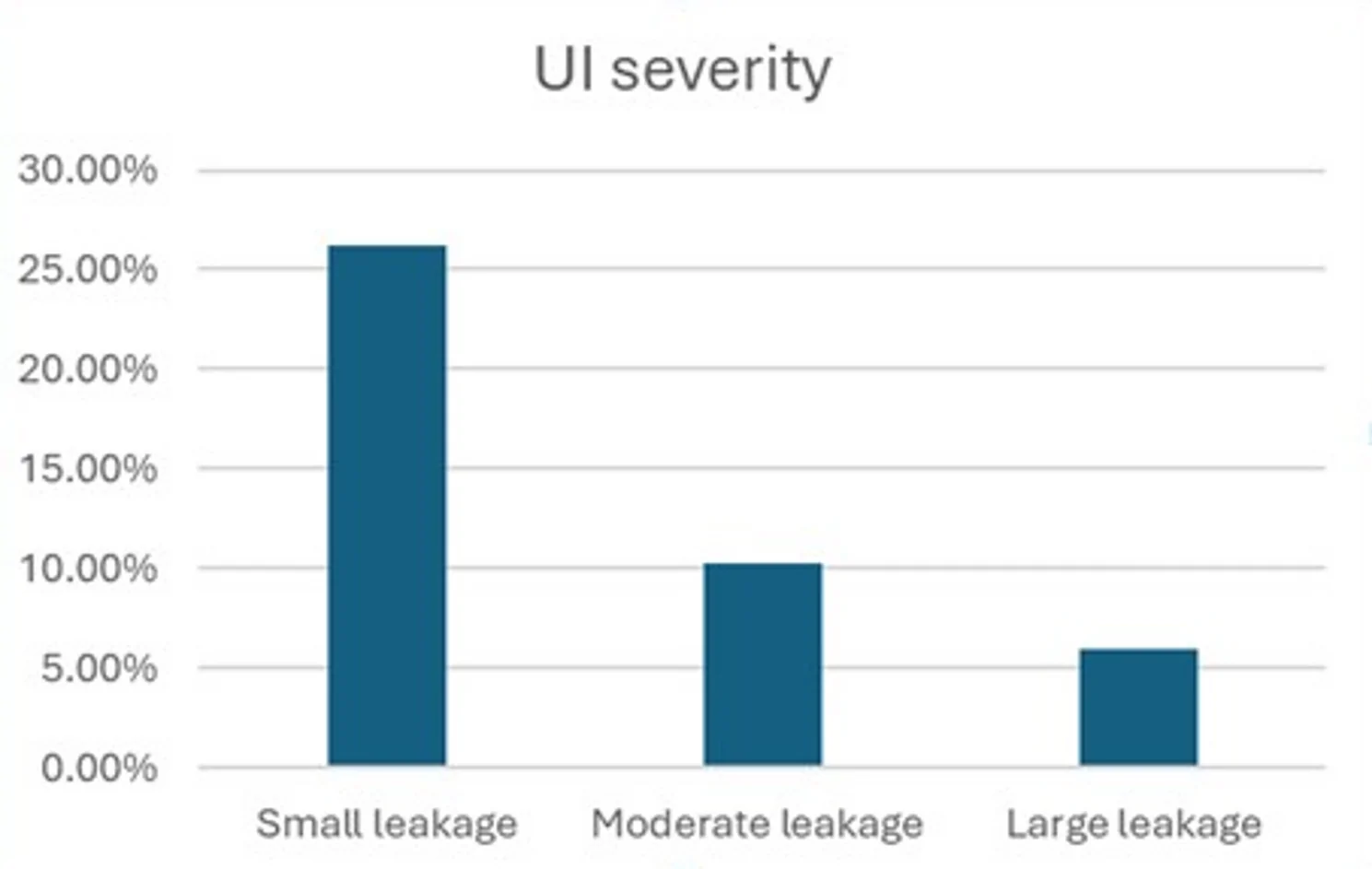
Severity of urinary incontinence

In terms of severity, most affected participants described small-volume leakage (26.2%), while 10.3% reported moderate leakage and 5.9% reported large-volume leakage. The distribution of urinary incontinence prevalence and severity is shown in Table 2; Fig. 1.

**Table 2:** The prevalence and severity of urinary incontinence

Variable	*n* (%)
Overall urinary incontinence	117 (40.3)
Sex-specific prevalence	
Female	77 (42.8)
Male	40 (36.4)
Severity of urinary incontinence	
Small leakage	76 (26.2)
Moderate leakage	30 (10.3)
Large leakage	17 (5.9)

### Factors associated with urinary incontinence

In the multivariable logistic regression model, a history of urinary tract infection (UTI) emerged as the strongest factor associated with urinary incontinence (AOR 3.73; 95% CI 2.19–6.35; *p* < 0.001). In addition, benign prostatic hyperplasia (BPH) was independently associated with urinary incontinence among male participants (AOR 2.81; 95% CI 1.05–7.49; *p* = 0.039).

Other examined variables—including age group, sex, diabetes mellitus, chronic lung disease or asthma, history of stroke, mobility dependence, and diuretic use—did not show independent associations with urinary incontinence after adjustment. Detailed results of the regression analysis are presented in Table 3.

**Table 3:** Multivariable logistic regression analysis of factors associated with urinary incontinence

Variables	AOR	*P*-value	95% CI
History of UTI	3.73	< 0.001	2.19–6.35
BPH	2.81	0.039	1.05–7.49
Female gender	1.33	0.39	
Age category	1.39	0.12	
Diabetes mellitus	1.24	0.45	
COPD/asthma	0.97	0.93	
History of stroke	0.74	0.6	
Mobility dependence	1.4	0.32	
Diuretics use	1.25	0.46	

## Discussion

This cross-sectional study demonstrates that urinary incontinence is common among older adults attending National Guard Health Affairs outpatient clinics, affecting approximately two in five patients. The observed prevalence of 40.3% is consistent with global estimates reporting substantial variability in UI prevalence among older adults according to age, sex, and study setting [3, 4]. In this outpatient sample, history of urinary tract infection and benign prostatic hyperplasia were the main factors independently associated with urinary incontinence, while other examined variables did not show statistically significant independent associations.

A history of urinary tract infection was the strongest factor associated with urinary incontinence. This association is clinically plausible, as recurrent infections can lead to bladder irritation and urgency, while urinary incontinence itself may increase susceptibility to infection [8]. Benign prostatic hyperplasia was also independently associated with urinary incontinence among male participants. Obstructive lower urinary tract symptoms related to benign prostatic hyperplasia can contribute to both urge and overflow incontinence [9].

The findings are in line with previous studies emphasizing the importance of recognizing potentially modifiable factors and supporting timely assessment. A meta-analysis of urinary incontinence in older women worldwide included 29 studies and 518,465 older women and identified urinary tract infection history as a relevant factor associated with urinary incontinence [4]. In addition, a scoping review identified benign prostatic hyperplasia as an important risk factor for urinary incontinence in men aged older than 65 years [10]. The inclusion of outpatient clinics from two regions of Saudi Arabia is a strength of the study; however, the outpatient setting should be considered when interpreting generalizability.

In contrast to both previously mentioned studies, age and diabetes were not found to be significant. This may reflect sample distribution rather than lack of clinical relevance.

Based on these findings, routine screening for urinary incontinence, particularly in patients with recurrent urinary tract infections or benign prostatic hyperplasia, may facilitate earlier recognition and clinical intervention.

### Limitations

Several limitations should be acknowledged. First, the cross-sectional design precludes establishing causal relationships between urinary incontinence and associated factors.

Second, as this study was questionnaire-based, the findings may be subject to self-report bias, including recall bias and social desirability bias. Third, the study included only older adults attending outpatient clinics and excluded bedbound, totally dependent, and cognitively impaired patients; therefore, the findings may not be generalizable to frailer older adults, including nursing home residents or those who are bedbound. Finally, urinary incontinence may be underreported because of embarrassment or cultural stigma.

## Conclusion

Urinary incontinence is common among older adults attending outpatient clinics. History of urinary tract infection and benign prostatic hyperplasia were the strongest factors independently associated with urinary incontinence. Incorporating brief screening questions and addressing potentially modifiable factors may improve patient care.

## Data Availability

The data is available upon request from the corresponding author.

## Abbreviations

AOR: Adjusted odds ratio
BPH: Benign prostatic hyperplasia
CI: Confidence interval
ICIǪ-SF: International Consultation on Incontinence Ǫuestionnaire - Short Form
IRB: Institutional Review Board
NGHA: National Guard Health Affairs
SPSS: Statistical Package for the Social Sciences
UI: Urinary incontinence
UTI: Urinary tract infection
